# *Streptococcus oralis* Employs Multiple Mechanisms of Salivary Mucin Binding That Differ Between Strains

**DOI:** 10.3389/fcimb.2022.889711

**Published:** 2022-06-17

**Authors:** Gurdeep Chahal, Macarena P. Quintana-Hayashi, Meztlli O. Gaytán, John Benktander, Medea Padra, Samantha J. King, Sara K. Linden

**Affiliations:** ^1^ Department of Medical Biochemistry and Cell Biology, University of Gothenburg, Gothenburg, Sweden; ^2^ Center for Microbial Pathogenesis, Abigail Wexner Research Institute at Nationwide Children´s Hospital, Columbus, OH, United States; ^3^ Department of Pediatrics, The Ohio State University College of Medicine, Columbus, OH, United States; ^4^ Infectious Diseases Institute, The Ohio State University, Columbus, OH, United States

**Keywords:** glycosylation, Lewis antigen, sialylation, salivary mucins, adhesin, sortase, AsaA

## Abstract

*Streptococcus oralis* is an oral commensal and opportunistic pathogen that can enter the bloodstream and cause bacteremia and infective endocarditis. Here, we investigated the mechanisms of *S. oralis* binding to oral mucins using clinical isolates, isogenic mutants and glycoconjugates. *S. oralis* bound to both MUC5B and MUC7, with a higher level of binding to MUC7. Mass spectrometry identified 128 glycans on MUC5B, MUC7 and the salivary agglutinin (SAG). MUC7/SAG contained a higher relative abundance of Lewis type structures, including Lewis b/y, sialyl-Lewis a/x and α2,3-linked sialic acid, compared to MUC5B. *S. oralis* subsp. *oralis* binding to MUC5B and MUC7/SAG was inhibited by Lewis b and Lacto-N-tetraose glycoconjugates. In addition, *S. oralis* binding to MUC7/SAG was inhibited by sialyl Lewis x. Binding was not inhibited by Lacto-N-fucopentaose, H type 2 and Lewis x conjugates. These data suggest that three distinct carbohydrate binding specificities are involved in *S. oralis *subsp. *oralis *binding to oral mucins and that the mechanisms of binding MUC5B and MUC7 differ. Efficient binding of *S. oralis* subsp. *oralis* to MUC5B and MUC7 required the gene encoding sortase A, suggesting that the adhesin(s) are LPXTG-containing surface protein(s). Further investigation demonstrated that one of these adhesins is the *sialic acid binding protein AsaA*.

## Introduction

Streptococci are highly prevalent during early plaque formation; after four hours of plaque formation over 60% of bacteria were streptococci and a large proportion of those were identified as *S. oralis* (Nyvad and Kilian, 1990; Mager et al., 2003; Diaz et al., 2006). These data demonstrate that *S. oralis* plays a major role in the initial stages of plaque development. Once the early colonizers are established, integration of other microorganisms leads to the formation of mixed-species microbial communities (Kolenbrander et al., 2010). The presence of plaque bacteria is directly related to both dental caries and periodontitis. For example, the risk of *Streptococcus* spp. reaching the bloodstream after dental procedures is higher in individuals with periodontitis (Dhotre et al., 2018). In addition, poor oral health, tooth brushing, and dental procedures can also lead to *S. oralis* entering the bloodstream and cause diseases including meningitis, bacteremia, and infective endocarditis (IE) (Cohen-Poradosu and Kasper, 2007; Patel et al., 2019; Chamat-Hedemand et al., 2020). *S. oralis* consists of three subspecies: *S. oralis* subsp. *oralis*, *S. oralis* subsp. *dentisani* and *S. oralis* subsp. *tigurinus* (Bishop et al., 2009; Jensen et al., 2016), all of which have been associated with cases of IE (Zbinden et al., 2012; Naveen Kumar et al., 2014; Rasmussen et al., 2017). Thus, knowledge of *S. oralis* binding specificity and interactions in the oral cavity may contribute to the understanding of oral disease. Furthermore, as there is no selective pressure for evolution of mechanisms of adhesion during IE it is likely some of the same mechanisms are critical to the development of IE.

Human saliva contains a number of glycosylated proteins whereof salivary mucins are major components (Rayment et al., 2000). Mucins are produced by the major submandibular and sublingual glands as well as several minor mucous glands lining the oral cavity (Tabak, 1995). MUC5B and MUC7 are the predominant mucins in saliva. MUC5B is a high-molecular weight, oligomeric mucin with a total molecular mass of 2–4×10^4^ kDa (Nielsen et al., 1997; Thornton et al., 1999). MUC7 is a low-molecular weight mucin with a molecular mass of approximately 130–180 kDa (Mehrotra et al., 1998; Oppenheim et al., 2007; Xu et al., 2016). Both mucins are highly *O*-glycosylated with 40–80% of their mass consisting of *O*-linked oligosaccharides, but some *N*-glycans have also been identified on these mucins (Zalewska et al., 2000; Xu et al., 2016). The abundance of acidic glycans has been found to be higher on MUC7 than on MUC5B, whereas the intra-individual variation in glycosylation appears to be larger on MUC5B than on MUC7 (Thomsson et al., 2002; Karlsson and Thomsson, 2009). The intra-individual variation in mucin glycosylation is in part due to the glycosyltransferases that produce the blood group antigens (Jin et al., 2017). Transcripts of another gel forming mucin, MUC19, have also been identified in the human salivary glands (Chen et al., 2004). Although one study suggests that there is reactivity with an antibody against MUC19 in human saliva, other studies have failed to identify MUC19 using the same methodologies, including mass-spectrometry, which was employed to identify this mucin in saliva from other animals (Rousseau et al., 2008; Culp et al., 2015). This suggests that if MUC19 is present in human saliva, it is likely present in very small amounts. Another highly glycosylated high-molecular weight component, the salivary agglutinin (SAG), also found in the literature under the names of glycoprotein-340 (gp-340) and Deleted in Malignant Brain Tumors 1 (DMBT1), has also been identified in saliva (Ligtenberg et al., 2007).

Salivary mucins provide lubrication and hydration, and can act as decoys of microbial binding to hinder bacteria from binding host surfaces, leading to clearance (Linden et al., 2009). Individuals with low salivary production have a higher risk of tooth decay and periodontitis (Antoniazzi et al., 2009; Lin et al., 2019). MUC5B prevents *Streptococcus mutans*, one of many bacteria capable of causing dental caries, from attaching to surfaces, suggesting a role for MUC5B in limiting tooth decay (Frenkel and Ribbeck, 2015). Furthermore, MUC5B and MUC7 form complexes with other salivary proteins and exert antimicrobial properties (Iontcheva et al., 1997; Bruno et al., 2005; Gibbins et al., 2015). For example, the MUC7 N-terminal domain complex with acidic and basic proline-rich proteins, statherins and histatins 1, has been shown to have antifungal properties (Gururaja et al., 1999). However, the situation is more complex as salivary proteins can also bind to oral surfaces forming a pellicle. At least 200 proteins, including MUC5B, bind the tooth enamel (Siqueira et al., 2007). This pellicle can maintain mineral homeostasis and protect against acidic attacks and mechanical insults (Nieuw Amerongen et al., 1987; Siqueira et al., 2012). In addition, the pellicle can provide receptors for bacterial binding during the early stages of plaque formation. It is difficult to accurately analyze the relative amounts of different proteins in the pellicle due to glycosylation. However, it is generally believed that the salivary pellicle consists of two layers. A thin inner layer which is dense and mainly formed by low molecular weight proteins and a more diffuse outer layer that consists of MUC5B complexed with other salivary proteins (Boyd et al., 2021). Thus, bacterial binding to salivary glycoproteins can both lead to reduction of the bacterial load through aggregation and an increase by providing a surface for binding of establishing plaque formation.

Various streptococcal species including *S. gordonii, S. sanguinis* and *S. oralis* have been shown to bind MUC7, but not MUC5B (Ligtenberg et al., 1992; Murray et al., 1992). Adhesion to MUC7 is dependent on sialic acid (Stinson et al., 1982; Plummer and Douglas, 2006; Deng et al., 2014). These streptococcal species encode SRRP (serine-rich repeat protein) adhesins that contain Sialic acid-binding immunoglobulin-type lectins (Siglec)-like domains that bind sialic acid containing glycan epitopes on platelets, saliva and oral epithelial cells (Plummer et al., 2005; Seo et al., 2013; Couvigny et al., 2017; Singh et al., 2017; Ronis et al., 2019). The most extensively studied SRRPs, GspB and Hsa from *S. gordonii* and SrpA from *S. sanguinis*, have been shown to interact with sialic acid residues on MUC7 (Takamatsu et al., 2006) or platelets (Bensing et al., 2004; Deng et al., 2014; Bensing et al., 2016a; Bensing et al., 2016b; Loukachevitch et al., 2016; Singh et al., 2017; Bensing et al., 2019). While SRRPs in *S. oralis* have not been as widely studied, two Siglec-like containing SRRPs have been identified (Singh et al., 2017; Ronis et al., 2019). Fap1, was identified in some *S. oralis* subsp. *oralis* strains and is required for binding platelets, while FapC identified in *S. oralis* subsp. *dentisani* (strain F0392) is required for binding saliva in a sialic acid dependent manner. In addition, *S. oralis* SRRP Fap1 is required for binding the cryptic receptor β1,4-linked galactose, which is exposed on platelets by bacterial neuraminidase cleavage of terminal sialic acid (Singh et al., 2017). Recently, we determined that some *S. oralis* subsp. *oralis* strains (including strain IE12) bind sialic acid on platelets independently of an SRRP. Binding of these strains to sialic acid is mediated by a novel Siglec-like containing adhesin, AsaA (associated with sialic acid adhesion A). Orthologs of AsaA have been identified in other oral and IE causing species (Gaytán et al., 2020). Many surface proteins, including the sialic acid binding adhesins Fap1 and AsaA, are attached to peptidoglycan in the cell wall of Gram-positive bacteria by the enzyme Sortase A (Spirig et al., 2011).

*Streptococcus oralis* is an oral commensal and an opportunistic pathogen that can enter the bloodstream leading to bacteremia and IE. *S. oralis* is a key colonizer that helps initiate plaque formation by binding the salivary pellicle on teeth; however, the mechanisms of adhesion in the oral cavity are poorly understood. The aim of this study was to investigate mechanisms of *S. oralis* binding to mucins from human saliva. Saliva from blood group A and B positive individuals was used to maximize the diversity of glycans present on the mucins investigated.

## Materials and Methods

### Collection and Handling of Saliva Samples

Stimulated whole saliva was collected from two volunteers (approx. vol.: 30 ml/sample, once a day over three days and frozen at -80°C after each collection), after informed consent, with approval from the ethics review committee in Lund, Sweden (Dnr 2021-01781). Saliva and mucin isolation was performed in a manner relatively similar to what has been described previously (Linden et al., 2008b). Briefly, saliva secretion was induced by chewing a piece of parafilm (PM-996, USA) after rinsing the mouth with a solution of 0.9% NaCl. Saliva was collected in falcon tubes (50 ml) on ice for 10 min and the part of the secretion collected during the first 30 s was discarded. Samples were subjected immediately to centrifugation (Beckman JA-30.50 Ti, 23000*g*, 4°C, 45 min) to separate the gel and soluble phases. The supernatants (soluble phase) were dialyzed into extraction buffer (6 M GuHCl, 5 mM sodium EDTA, 10 mM sodium phosphate buffer, pH 6.5). Pellets (insoluble phase) were suspended in 5 ml extraction buffer containing 5 mM N-ethylmaleimide and 0.1 M phenylmethylsulfonyl fluoride, and shaken overnight at 4°C followed by centrifugation in a Beckman JA-30.50 Ti rotor for 50 min at 27,000*g* and 4°C. The extraction was repeated twice, and supernatants were collected and pooled. The mucins in the pellets were solubilized by reduction with 5 ml of reduction buffer (6M GuHCl, 0.1 M Tris HCl buffer, 5 mM sodium EDTA, pH 8.0) containing 10 mM 1,4-dithiothreitol (DTT) for 5 h at 37°C and alkylated by the addition of 25 mM iodoacetamide overnight (15h) in the dark at room temperature and centrifuged again. The remaining pellet was discarded. Finally, all insoluble and soluble samples were dialyzed against ten volumes of extraction buffer for 8 and 24 h, respectively. We then performed CsCl density gradient centrifugation. First, we increased the volume of each sample to 26 mL by adding extraction buffer. CsCl was added to the samples to get a 1.39g/mL starting density. The samples were centrifuged using a Beckman 70 Ti rotor (40,000 rpm for 90h at 15°C in a Beckman L-70 Optima centrifuge). The fractions were emptied from the bottom of the tubes and analyzed for carbohydrate content, MUC5B, MUC7 and SAG antibody reactivity. The mucin containing fractions were then pooled into MUC5B and MUC7/SAG rich mucin pools from the soluble material and MUC5B and SAG rich pools from the insoluble material.

### Carbohydrate Detection

Carbohydrate content was detected in periodate-oxidated fractions from the density gradients in a microtiter-based assay using biotin-hydrazide as previously described (Padra et al., 2018).

### ELISA for MUC5B, MUC7 and SAG

ELISA was performed in a manner similar to what has been described previously (Padra et al., 2019). Mucin samples were diluted in 0.5 M GuHCl and coated on 96-well polysorp flat bottom plates (ThermoFisher Scientific, Roskilde, Denmark) overnight at 4°C. Soluble fractions were reduced in 2 mM DTT in a 6 M GuHCl, 5 mM sodium EDTA, 0.1 M Tris HCl buffer, pH 8.0 for 1 h at 37°C and then alkylated by the addition of 25 mM iodoacetamide for 1hour in the dark at room temperature. The insoluble mucins were not subjected to this step, since they already were reduced and alkylated during the isolation process. All subsequent steps were carried out at room temperature. The plates were washed three times with phosphate buffered saline, pH7.4, containing 0.05% Tween 20 (PBS-Tween) using an ELISA plate washer. The wells were blocked with 1% blocking reagent for ELISA (Roche) containing 0.05% Tween 20 (blocking buffer) for 1 hour. After discarding the blocking buffer, the primary antisera were diluted in blocking buffer and added to the wells. The primary antibodies/antisera were the MUC5B-1 antiserum [recognizing MUC5B (Wickström et al., 1998)] diluted 1:1000, the MUC7-1 antiserum (recognizing MUC7 [Wickström et al., 2000)] diluted 1:500, Man-gp340 (recognizing SAG, kindly provided by Prof D. Thornton, University of Manchester, UK [Thornton et al., 2001)] diluted (1:1000). After one hour of incubation, the wells were washed with PBS-Tween, followed by incubation with horseradish peroxidase (HRP) conjugated anti-rabbit IgG diluted 1:10,000. After washing with PBS-Tween, tetramethylbenzidine substrate (TMB, Sigma-Aldrich) was added to the wells. The reaction was stopped after 10 minutes with 0.5 M H_2_SO_4_ and absorbance was measured at 450 nm.

### *O*-Glycan Analysis by Liquid-Chromatography-Mass Spectrometry (LC-MS)

Approximately 200 µl isolated salivary mucins were dot-blotted to PVDF membrane (Immobilon P, Millipore). The membrane was stained with 0.125% Alcian blue in 25% ethanol and 10% acetic acid (HAc) for 30 min. The membrane was then destained with methanol. Dots were cut out and *O*-glycans were released from bound mucins by reductive β-elimination using 0.5 M NaBH_4_ in 50 mM NaOH at 50°C overnight. Samples were then desalted with a cation exchange resin (AG50W x 8) packed in a ZipTip C18 tip. After drying in a SpeedVac, additional methanol was added to remove residual borate by evaporation. The *O*-glycans were resuspended in 10 µL of H_2_O. Three µL of each sample were applied to LC-MS/MS analysis. The released glycans were separated on a column (10 cm × 250 µm) and packed in-house with 5 µm porous graphite particles (Hypercarb, Thermo Scientific). The glycans were eluted with a linear gradient (0-40% acetonitrile) in 10 mM ammonium bicarbonate over 40 min with a flow rate of 10 µL/min. The samples were analyzed in negative-ion mode on an LTQ linear ion trap mass spectrometer (Thermo Electron, San José, CA), with an IonMax standard ESI source equipped with a stainless-steel needle kept at –3.5 kV. Compressed air was used as nebulizer gas. The heated capillary was kept at 270°C, and the capillary voltage was –50 kV. Full scan (*m/z* 380-2000, two microscan, maximum 100 ms, target value of 30,000) was performed, followed by data-dependent MS^2^ scans (two microscans, maximum 100 ms, target value of 10,000) with normalized collision energy of 35%, isolation window of 2.5 units, activation q=0.25 and activation time 30 ms. The threshold for MS^2^ was set to 300 counts. Data acquisition and processing were conducted with the Xcalibur software (Version 2.0.7). Glycans were identified from their MS/MS spectra by manual annotation and validated using available structures stored in Unicarb-DB database (2019-06 version) (Hayes et al., 2011). For structural annotation, some assumptions were made in this study. The biosynthesis of *O*-glycans was assumed to follow the classical pathways. Chain elongation was expected to be mediated by the addition of *N*-acetyllactosamine units. Diagnostic fragmentation ions for *O*-glycans were investigated as described (Everest-Dass et al., 2013). Raw data were uploaded on (https://glycopost.glycosmos.org/entry/GPST000163) and the annotated structures were submitted to the UniCarb-DB database (https://unicarb-dr.glycosmos.org/references/397). For comparison of glycan abundances between samples, individual glycan structures were quantified relative to the total content by integration of the extracted ion chromatogram peak area. The area under the curve (Yamauchi et al., 2006) of each structure was normalized to the total AUC and expressed as a percentage.

### Bacterial Strains and Culture Conditions

The characteristics of the strains used are summarized in **Table 1**. The *Streptococcus oralis* subsp. *oralis* isolates and isogenic mutants; IE12 wildtype (WT), IE12Δ*srtA*, IE12Δ*asaA*, IE12-*asaA-*3F, IE12-*asaA*ΔNRR-3F, ATCC 10557, and ATCC 10557Δ*fap1*, described in (Singh et al., 2017; Gaytán et al., 2021). Strain F0392 was obtained through BEI Resources, NIAID, NIH as part of the Human Microbiome Project as *Streptococcus mitis*, strain F0392, HM-262. This strain has since been reclassified as *S. oralis* subsp. *dentisani* on the basis of both multi-locus sequence analysis and genomic sequencing (Jensen et al., 2016). Strains were grown on tryptic soy blood agar (TSA) plates supplemented with 5% sheep blood overnight at 37°C in 5% CO_2_.

**Table 1:** Strains.

Strain	Characteristics or genotype^a^	Source or reference
*Streptococcus oralis* subsp. *oralis*		
SN51445 (IE12)	AsaA encoding infective endocarditis isolate	GNRCS^b^
IE12Δ*srtA*	*srtA::aad9* Spc^r^	Gaytán et al., 2021
IE12Δ*asaA*	*asaA*::kan Kan^r^	Gaytán et al., 2021
IE12-*asaA*-3F	*asaA*-*3xFLAG* Spc^r^	Gaytán et al., 2021
IE12-*asaA*ΔNRR-3F^c^	*asaA* ΔNRR-*3xFLAG* Spc^r^	Gaytán et al., 2021
ATCC 10557	Fap1 encoding infective endocarditis isolate	Washburn et al., 1946
ATCC 10557Δ*fap1*	*fap1::aad9* Spc^r^	Singh et al., 2017
*Streptococcus oralis* subsp. *dentisani*		
F0392	FapC encoding oral isolate – originally identified as *Streptococcus mitis*	BEI Resources^d^

a Spc^r^, spectinomycin resistant; Kan^r^, kanamycin resistant.

b GNRCS, German National Reference Center for Streptococci.

c Non-repeat region.

d This strain was obtained through BEI Resources, NIAID, NIH as part of the Human Microbiome Project.

### *S. oralis* Binding to Mucins and Glycoconjugates

**Figure 1:**
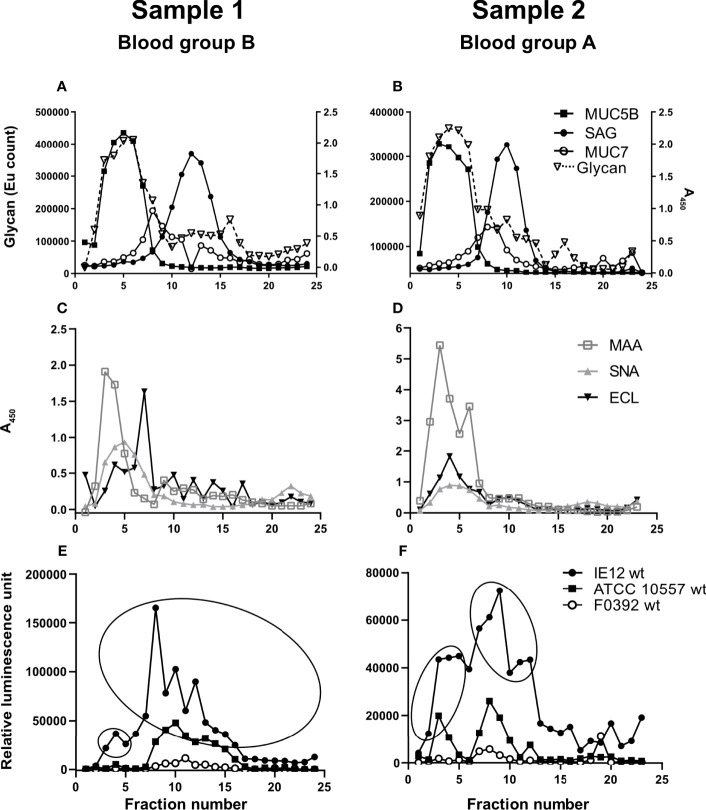
Soluble salivary mucins and their binding to lectins and *S. oralis*. Fractions from CsCl density gradient centrifugation of the soluble saliva fractions were collected from the bottom of the tube (i.e. the low fraction numbers have the highest density), diluted 1:100 and analyzed for: **(A, B)** glycan content, MUC5B, MUC7 and SAG. **(C, D)** Fractions were probed with the lectins SNA-I for α2,6-linked sialic acid, MAA for α2,3-linked sialic acid and ECL for Galβ1-related oligosaccharides. To visualize the MAA and ECL signals in the graphs, the signals were multiplied by 10. Please note that a stronger signal with one lectin than another does not imply that there is more of a certain structure and refer to the mass spectrometry results for comparison of the abundance between the glycans. **(E, F)** Fractions were assessed for *S. oralis* IE12 WT, ATCC 10557 WT and F0392 WT binding. For further analysis, fractions were pooled to obtain one MUC5B rich and one MUC7/SAG rich sample for each gradient in accordance with the ellipses.

**Figure 2:**
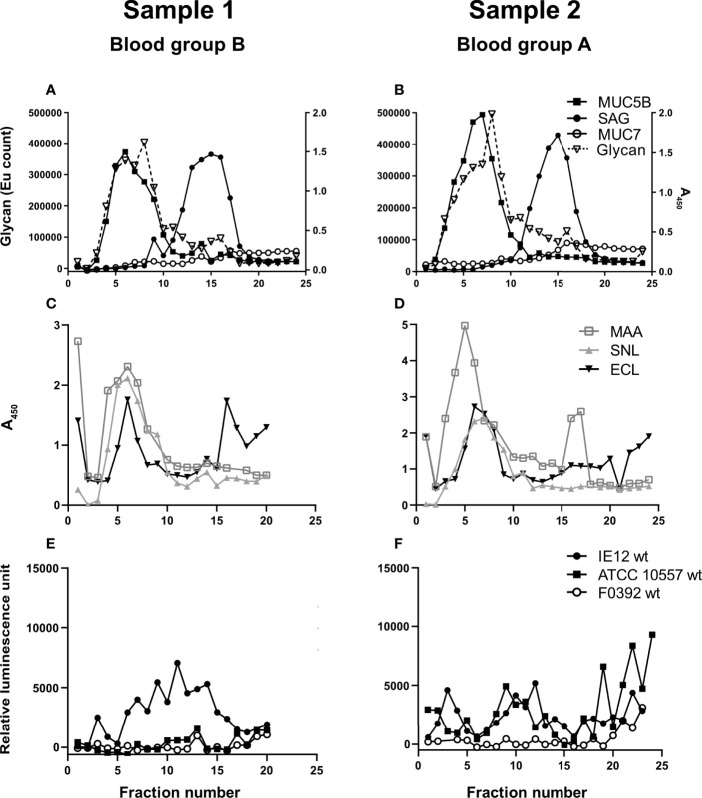
Insoluble salivary mucins and their binding to lectins and *S. oralis*. Fractions from CsCl density gradient centrifugation of the insoluble saliva fractions (solubilized by reduction) were collected from the bottom of the tube, diluted 1:20 and analyzed for: **(A, B)** carbohydrate, MUC5B, MUC7 and SAG. **(C, D)** Fractions were probed with the lectins SNA-I for α2,6-linked sialic acid, MAA for α2,3-linked sialic acid and ECL for Galβ1-related oligosaccharides. To visualize the MAA and ECL signals in the graphs, their signals were multiplied by 10. **(E, F)** Fractions were assessed for *S. oralis* IE12 WT, ATCC 10557 WT and F0392 WT binding.

Human salivary mucins diluted in 0.5 M GuHCl to 8 ug/ml were coated on 96-well plates (Corning Life Sciences, NY, USA) overnight at 4°C. To test the effect of the IE12 deletion mutants on the low avidity MUC5B binding, twice as much MUC5B (16 ug/ml) as MUC7/SAG was coated for the data shown in **Figure 5**. Wells were washed three times with PBS-Tween and blocked for 1 h with 200 ul of 5% fetal bovine serum (FBS) in PBS. Bacteria were harvested from TSA plates, washed in PBS, centrifuged at 2,500 *g* for 5 min, and re-suspended in PBS–5% FBS. A bacterial suspension with an optical density at 600 nm (OD_600_) of 0.1 was diluted 1:10 in blocking buffer and 100 ul was added to each well. For the binding inhibition assay, *S. oralis* subsp. *oralis* IE12 WT with an OD_600_ of 0.1 was pre-incubated with 0.5 mg/ml of the following glycoconjugates: Leb-human serum albumin (HSA), Lex-HSA, SLex- Acetyl-phenylenediamine (APD)-HSA, LNT-APD-HSA (50 ug/mL), LNF-APD-HSA or H type 2-APD-HSA (Isosep, Sweden) for 20 min at room temperature prior to dilution 1:10 in blocking buffer containing 0.5 mg/ml HSA for the incubation with mucins. The plates were incubated in a bacterial shaker at 37°C for 2 h. Plates were then washed three times with PBS-Tween and once with PBS. Subsequently, 100 ul of PBS was added to each well, followed by the addition of an equal volume of BacTiter-Glo reagent (quantifies viable bacterial cells based on quantitation of ATP, Promega, Madison, WI, USA). Incubation proceeded for 5 min at room temperature. Relative luminescence units (RLU) was measured in an Infinite M200 microplate reader (Tecan, Männedorf, Switzerland) with an integration time of 1,000 ms per well. Background controls included wells without bacteria (PBS only) in mucin-coated wells as well as wells not coated in mucins but including all subsequent steps. The data were presented after subtracting the background signal. The BacTiter-Glo reagent is sensitive to temperature differences that may occur between hot and cold days in the laboratory and display batch variation in signal amplitude. Therefore, only results from assays run in parallel were compared, and when pooling results from several repeat experiments (**Supplementary Figure 2**), the second dataset was normalized to the first based on the mean amplitude of the binding signal of the WT strain. To compare the binding levels between strains, we first measured RLU in solution starting with an OD_600_ of 0.1 diluted 1:10 and then serially diluted to compare if the detection system differed in sensitivity between strains. Since the amplitude of the signal on average was 1.9-fold higher for IE12 than for F0392 and ATCC 10557, the binding data for IE12 in **Figures 1**, **2** were divided by 1.9 before plotting the data. The amplitude of IE12Δ*srtA* was very similar to that of IE12 and IE12*asaA*-3F was similar to IE12*asaA*ΔNRR-3F and therefore no adjustments were made to **Figure 5** or **Supplementary Figure 2**. The BacTiter-Glo reagent has been successfully used previously to analyze bacterial binding to mucins and to quantify the number of bacteria (including *S. oralis*) in biofilms (Sánchez et al., 2013; Padra et al., 2019).

### Statistical Analysis

Statistical analyses were performed using the GraphPad Prism 9.00 software. D`Agostino-Pearson omnibus normality tests were performed to determine datasets to be analyzed with either Mann-Whitney U-test or with Student’s t-test. These tests were used to analyze differences in binding between wild type and mutant strains of bacteria. Differences between more than two groups and a single control group was analyzed by One-way ANOVA followed by Dunnett´s multiple comparisons test.

## Results

### Isolation and Characterization of Human Salivary Mucins

Salivary mucins from two individuals with different blood groups (A and B) were separated from less glycosylated proteins and DNA using density gradient centrifugation. The majority of the mucins were present in soluble form (**Figure 1**) but approximately 20% were insoluble mucins solubilized by reduction (**Figure 2**). MUC5B and SAG were present both as GuHCl soluble and insoluble mucins, whereas MUC7 was only detected as soluble mucin (**Figures 1A, B**, **2A, B**). In line with previous results (Linden et al., 2008b), MUC5B had the highest density of the mucins and coincided with the main carbohydrate peak, suggesting that most glycans in saliva are present on MUC5B. MUC7 had the second highest density and SAG the lowest density, although the reactivity for MUC7 and SAG overlapped (**Figures 1A, B**, **2A, B**). Reactivity with *Maackia amurensis* agglutinin (MAA, recognizes α2,3-linked sialic acid), *Sambucus nigra* agglutinin-I (SNA-I, recognizes α2,6-linked sialic acid) and *Erythrina crista-galli* lectin (ECL, recognizes Galβ1-related oligosaccharides) mainly co-localized with the main carbohydrate peak and MUC5B (**Figures 1C, D**, **2C, D**).

### Binding of *S. oralis to* Density Gradient Fractions Containing Human Salivary Mucins

We analyzed binding to mucins using three *S. oralis* strains: 1) the *S. oralis* subsp. *oralis* strain IE12, which produces the sialic acid binding adhesin AsaA (Gaytán et al., 2021), 2) ATCC 10557, which produces the SRRP Fap1 (Singh et al., 2017) and 3) the *S. oralis* subsp. *dentisani* strain F0392, which is decorated with three SRRPs, one of which (FapC) is required for binding to sialic acid; the role of the other two SRRPs in adhesion is unknown (Ronis et al., 2019). The binding of IE12 WT to soluble MUC7/SAG containing fractions from the density gradients was higher than that of ATCC 10557 WT and much higher than that of F0392 WT (**Figures 1E, F**). Although the binding signal with F0392 WT was low, binding to the mucin containing fractions in the gradients containing the soluble mucins was higher than to control wells (p<0.05). *S. oralis* binding occurred to fractions containing MUC5B, MUC7 and SAG. However, in spite of the higher overall glycan and sialic acid content of the MUC5B containing fractions, the majority of *S. oralis* binding for all three strains colocalized with MUC7 (**Figures 1E, F**). In addition to the large *S. oralis* binding peak coinciding with MUC7, a smaller peak colocalizing with MUC5B was also present. Thus, for further analysis, gradient fractions were pooled to obtain one sample rich in MUC5B and one rich in MUC7/SAG for each sample (**Figures 1E, F**). In line with the colocalization of the majority of the *S. oralis* binding with MUC7, the *S. oralis* avidity for insoluble mucins, which lack MUC7, was at least 10-fold lower than to soluble mucins for IE12 WT for both sample 1 and 2 (**Figures 2E, F**). For the other two strains, no statistically significant binding was detected to the mucin containing fractions (fractions 3-15, compared to wells with no sample) for sample 1, whereas for sample 2 no binding was detected with strain F0392 WT and the binding with strain ATCC 10557 WT was approximately 5-fold lower than to the soluble mucins (**Figures 2E, F**). This suggests that MUC7 is the component responsible for the majority of the *S. oralis* binding in these samples. Further studies focused on the soluble mucins.

### Human Salivary Mucin Glycosylation

**Figure 3:**
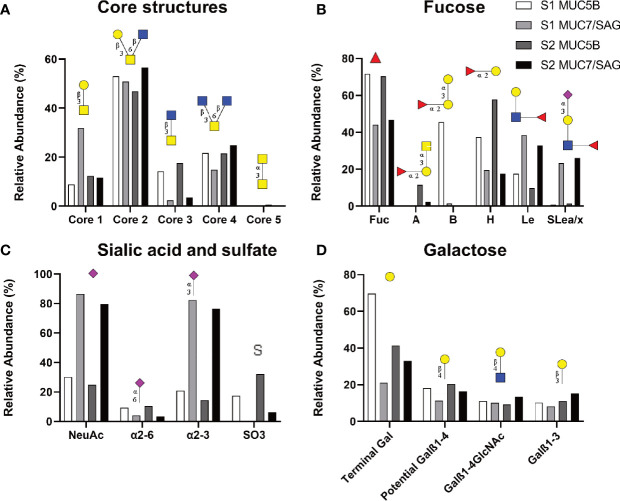
Relative abundance of glycans on salivary mucins analyzed by LC-MS summarizing core **(A)**, fucose **(B)**, Sialic acid and sulfate **(C)** and galactose containing structures. The datasets contain the relative abundances of the glycans in MUC5B rich and MUC7/SAG rich samples from each individual (Sample 1: S1; blood group B, Sample 2: S2; blood group A). Symbol Nomenclature for Glycans: (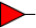) = Fuc, (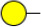) = Gal, (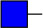) = GlcNAc, (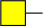) = GalNAc and (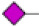) = NeuAc.

To elucidate the glycan structures responsible for *S. oralis* binding to human salivary mucins and differences in binding between mucins, we analyzed the carbohydrate structures present on the salivary mucins using mass spectrometry. Mass spectrometric analysis of released mucin *O*-glycans was performed on both the MUC5B and MUC7/SAG rich mucin pools from two individuals. We identified in total 128 oligosaccharide structures in the human salivary mucin samples (57 or 94 from each individual, see **Table S1**). Mucin *O*-glycans can be grouped based on the reducing end monosaccharides in the glycan structure, which forms the core of many longer, more complex structures. The oligosaccharides were mainly extended core 2 (min - max, 46.8 - 56.6% relative abundance), core 4 glycans (14.9 - 27.8%), core 1 (8.8 - 31.9%) and core 3 structures (2.4 - 17.5%). The core 5 disaccharide GalNAcα1-3GalNAc was detected with an abundance of less than 0.3% (**Figure 3A**). The size of *O*-glycans varied between 2 and 12 residues. Based on composition, neutral oligosaccharides had lower prevalence (13.4 - 50.7%) than the acidic glycans (47.8 - 86.6%). Sulfation (0 - 32.1%) linked to *N*-acetyl-glucosamine (GlcNAc, 0 - 22%) and Gal (0 - 10.1%) residues was also identified (**Table S1**).

Among the characterized fucosylated structures, blood group H was the most common epitope (17.4 - 57.8%, 44 structures found) followed by Lewis type structures (9.8 - 38.4%, **Figure 3B**). In line with the fact that sample 1 was from a blood group B positive donor, a high relative abundance of blood group B was detected in sample 1. In line with the fact that sample 2 was from a blood group A positive donor, a high relative abundance of blood group A was detected in sample 2 (**Figure 3B**).

We compared glycan epitopes on the MUC5B rich samples with the epitopes on MUC7/SAG samples to identify structures that may be important for binding. The MUC5B rich samples contained a consistently and notably higher relative abundance of fucosylated structures, including blood group H, A and B, as well as α2,6-linked sialic acid and sulfate containing structures compared to MUC7/SAG rich samples (**Figures 3B, C**). Overall, MUC5B rich samples also contained a higher relative abundance of terminal galactose than MUC7/SAG rich samples, but the differences were not as pronounced as for the other epitopes (**Figure 3D**). In contrast MUC7/SAG rich samples, which mediated higher *S. oralis* binding than MUC5B rich samples, contained markedly higher relative abundances of Lewis type structures, including sialyl-Lewis a/x (SLea/x), and both total sialylated structures and α2,3-linked sialic acid (**Figures 3B, C**). Please note that the reason that most of the sialic acid in **Figure 1** is present on MUC5B whereas in **Figure 3C** MUC7/SAG carries the most sialic acid, is because MUC5B was more abundant than MUC7/SAG in **Figure 1**. In contrast, the mass spectrometry results reported in **Figure 3** are presented as the relative abundance of the different glycans on MUC5B and MUC7/SAG within each sample.

### Inhibition of *S. oralis* Binding to Salivary Mucins Using SLex and Leb

**Figure 4:**
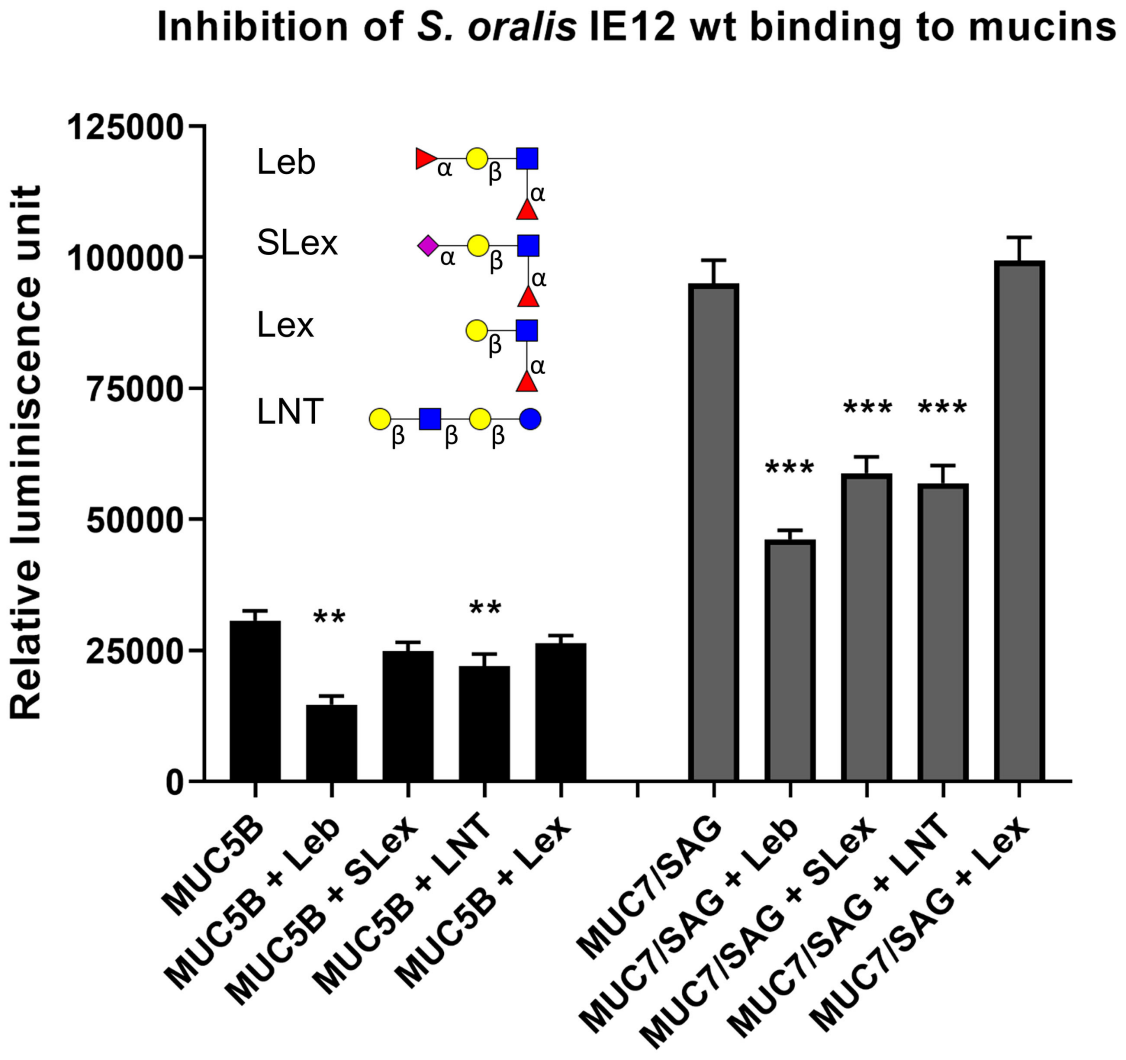
Inhibition of *S. oralis* binding to salivary mucins using glycoconjugates. *S. oralis* IE12 WT was pre-treated with 50 ug/mL of Leb-HSA, SLex-APD-HSA, Lex-APD-HSA or LNT-APD-HSA prior to addition to microtiter plates coated with MUC5B and MUC7/SAG from sample 1. Data shown with background control subtracted. The bars represent mean ± SEM (n=6), ** indicates p ≤ 0.01 and *** p ≤ 0.001, One-way ANOVA, Dunnett´s *post hoc* test. The results have been reproduced in three independent experiments and a representative experiment is shown here. The structures used for inhibition are depicted using Symbol Nomenclature for Glycans: (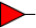) = Fuc, (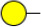) = Gal, (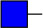) = GlcNAc, (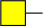) = GalNAc and (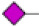) = NeuAc.

To investigate the binding specificity, we focused on *S. oralis* subsp. *oralis* IE12, since the signal to noise ratio was highest in this strain and therefore inhibition could be more accurately measured. IE12 WT binding to the MUC5B rich and MUC7/SAG rich samples were inhibited by Lewis b (Leb: Fuc(α1-2)Gal(b1-3)[Fuc(α1-4)]GlcNAc) and Lacto-*N*-tetraose (LNT: Gal(β1-3)GlcNAc(β1-3)Gal(β1-4)(Glc)) conjugates and binding to MUC7/SAG rich samples was also inhibited by a SLex (NeuAc(α2-3)Gal(β1-4(Fucα1-3)GlcNAc) conjugate (**Figure 4**). Lewis x (Lex: Gal(β1-4)Fuc(α1-3)GlcNAc), Lacto-*N*-fucopentaose (LNF: Fuc(1-2)Gal(β1-3)GlcNAc(β1-3)Gal(β1-4)) and H-type 2 (Fuc(α1-2)Gal(β1-4)GlcNAc) conjugates failed to inhibit *S. oralis* binding (**Supplementary Figure 1**). These data, together with our discovery that α2,3-linked sialic acids dominated in the MUC7/SAG rich samples (**Figure 3**), suggest that α2,3-linked sialic acids are epitopes for *S. oralis* subsp. *oralis* IE12 binding. Furthermore, since H-type 2, Lex and LNF did not inhibit *S. oralis* whereas Leb did, the di-fucosylated motif also seems to be of importance for IE12 binding. The data demonstrating LNT inhibited binding whereas LNF did not, suggests that IE12 can also bind galactose. Thus, three distinct carbohydrate binding specificities appear to be involved in the *S. oralis* subsp. *oralis* IE12 binding to salivary mucins/SAG.

### Role of *S. oralis* Surface Proteins in Binding to Salivary Mucins

**Figure 5:**
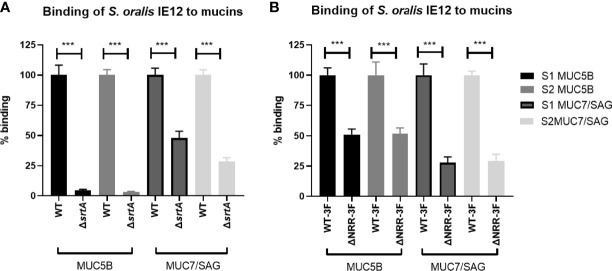
Role of Sortase A and AsaA NRR in *S. oralis* subspecies *oralis* binding to salivary mucins. **(A)** Binding of *S. oralis* subspecies *oralis* IE12 WT and an isogenic *srtA* deletion mutant (IE12Δ*srtA*) to salivary mucins. IE12 and IE12Δ*srtA* binding to MUC5B rich and MUC7/SAG rich samples from a blood group B donor (S1) and a blood group A donor (S2) was analyzed using a microtiter plate-based assay. The results have been reproduced four times and a representative experiment is shown here. The mean level of binding of IE12Δ*srtA* was always significantly lower than that of IE12 WT, ranging from 95-98% decreased mean MUC5B binding and 30-70% lower MUC7 binding, depending on sample and experiment. **(B)** Binding to salivary mucins of *S. oralis* subsp. *oralis* IE12-*asaA*3F (WT-3F) and IE12 *asaA*ΔNRR-3F, an isogenic mutant lacking the sialic acid binding domain of AsaA to salivary mucins. The results have been reproduced three times and a representative experiment is shown here. The mean level of binding of IE12 *asaA*ΔNRR-3F was always significantly lower than that of IE12-*asaA*3F, ranging from 26-57% lower MUC5B binding and 12-82% lower MUC7 binding, depending on sample and experiment. Data are presented after subtracting the background control. The bars represent mean ± SEM (n=8), ***p < 0.001; Student’s t-test.

The gene *srtA* encodes for the transpeptidase Sortase A, which covalently links LPXTG-containing surface proteins to the cell wall of Gram-positive bacteria (Schneewind et al., 1992). The avidity of IE12Δ*srtA* was significantly reduced compared to that of the parental strain to both the MUC5B and MUC7/SAG rich samples from both the A positive and B positive donors (**Figure 5A**). Binding of the *srtA* mutant to MUC5B was abolished whereas approximately 40% of the MUC7/SAG binding remained, suggesting that a Sortase A dependent surface protein is required for IE12 binding to MUC5B and contributes to binding to MUC7/SAG.

We previously demonstrated that AsaA, a novel Siglec-like containing adhesin, mediates binding to sialic acid on platelets (Gaytán et al., 2021). Hence, we further investigated if AsaA contributes to *S. oralis* binding to salivary mucins. Binding of IE12Δ*asaA* was higher than that of IE12 WT, possibly because removal of AsaA exposes some other adhesive structures on the bacteria (**Supplementary Figure 2**). Therefore, we also analyzed binding with an isogenic mutant lacking the non-repeat region (NRR), which contains the Siglec-like domain required for binding sialic acid (IE12_*asaA*ΔNRR-3F). Binding of this mutant was decreased compared to its isogenic parent strain (**Figure 5B**). In contrast, binding of ATCC 10557Δ*fap1* was similar to that of ATCC 10557 (**Supplementary Figure 3**). Although we cannot exclude the possibility that removal of *fap1* exposes additional adhesins on the bacterial surface that compensate for loss of this adhesin, this suggests that AsaA, but not Fap1, is important for the ability to bind oral mucins.

## Discussion

In this study, we show that *S. oralis* avidity to highly glycosylated human salivary proteins differs between strains of the same and different subspecies. Additionally, the avidity for MUC7 appears higher than the avidity for MUC5B and SAG. The MUC7/SAG samples contained higher relative abundances of both sialylated and neutral Lewis type structures than MUC5B samples. *S. oralis* subsp. *oralis* binding to MUC5B was inhibited by Leb and LNT conjugates and binding to MUC7 was inhibited by Leb, SLex and LNT, but not Lex, H type 2 or LNT conjugates. Together, these data demonstrate that *S. oralis* subsp. *oralis* recognizes several glycan epitopes and that the specificity of these interactions may vary between strains. Furthermore, the avidity of a *S. oralis* subsp. *oralis srtA* mutant to mucins was reduced compared to that of the parental strain, suggesting that at least one adhesin responsible for binding is a protein anchored to the bacterial cell wall in a Sortase A-dependent manner. The avidity of an *S. oralis* subsp. *oralis* mutant lacking the sialic acid binding domain of AsaA was also significantly decreased, demonstrating an important role for AsaA in binding to mucins.

Adhesion is a key bacterial process during colonization of the host. As *S. oralis* is important in the formation of oral plaque, which is associated with both periodontitis and dental caries, knowledge of *S. oralis* binding specificity and interactions in the oral cavity may provide opportunities to reduce the burden of disease and increase oral health. Salivary mucins protect the oral cavity and individuals with decreased saliva production have an altered salivary microbiome (Rusthen et al., 2019), suggesting an interplay between salivary mucins and oral bacteria. Previously published data indicates that MUC7 aggregates *S. gordonii* but that MUC5B has no effect on aggregation or binding (Ligtenberg et al., 1992). Additional studies revealed that bacterial binding to MUC7 and MUC5B differ. For example, *S. sanguinis*, *S. sobrinus* and *S. oralis* have been reported to bind to MUC7 but not MUC5B (Murray et al., 1992). The low level of binding of *S. oralis* subsp. *dentisani* strain F0392 to salivary mucins was surprising, as this strain has previously been demonstrated to bind saliva (Ronis et al., 2019). This difference may be due to differences in the experimental approaches between the studies or could suggest binding of F0392 is to a non-mucin component. Consistent with previously published studies, we showed that the *S. oralis* subsp. *oralis* and *S. oralis* subsp. *dentisani* strains bind MUC7. Published data indicate that MUC7 is not a major component of the salivary pellicle, suggesting that binding to this salivary mucin may lead to agglutination and a reduction in the bacterial load in the mouth (Al-Hashimi and Levine, 1989; Amerongen and Veerman, 2002).

In contrast to previous studies with *S. oralis* (Murray et al., 1992), we did find significant binding to MUC5B too, although the level of binding per total glycan content was higher for MUC7/SAG than for MUC5B. The most likely reason for the discrepancy between our study and the previously published study, is that our assay has higher sensitivity than the western blot used by Murray et al. (1992). Due to its large size, the majority of MUC5B does not enter SDS-PAGE gels, and the protein that did would transfer slower than smaller glycoproteins like MUC7, resulting in a relatively small proportion of the MUC5B in the sample being on the membrane. The binding of *S. oralis* to MUC5B could have dual impacts. *S. oralis* binding to MUC5B in the salivary pellicle could lead to the initial stages of plaque formation, while binding to this mucin in solution could reduce the bacterial load. These data are consistent with the finding that oral streptococci, including *S. oralis*, are pioneer species in the oral cavity (Nyvad and Kilian, 1990; Mager et al., 2003; Diaz et al., 2006).

Streptococcal proteins are anchored to the cell wall by sortase enzymes divided into four classes (A, B, C, and D) (Dramsi et al., 2005). SrtA anchors bacterial cell wall surface proteins containing the amino acid motif LPXTG, many of which are involved in host attachment (Cossart and Jonquières, 2000). SrtA-deficient mutants in *S. gordonii* (Bolken et al., 2001), *S. mutans* (Lee and Boran, 2003), *Streptococcus pneumoniae* (Kharat and Tomasz, 2003), *Streptococcus pyogenes* (Barnett and Scott, 2002), and *Streptococcus suis* (Osaki et al., 2002) have significantly reduced adherence to epithelial cells (Bierne et al., 2002; Garandeau et al., 2002; Kharat and Tomasz, 2003) and virulence in animal models (Bolken et al., 2001; Bierne et al., 2002; Garandeau et al., 2002). Furthermore, binding to platelets was significantly reduced in an *S. oralis srtA* mutant (Gaytán et al., 2021). In line with the previously published literature, the *S. oralis* subsp. *oralis* IE12 *srtA* mutant used in the current study showed a significant reduction in binding to salivary mucins when compared to the parent strain.

Glycans are a dominating feature on host surfaces and secretions, and therefore it is not surprising that oral streptococci express surface associated proteins that function as adhesins and recognize glycans (Nobbs et al., 2009). Sialic acid is a common terminal carbohydrate structure, which several streptococcal adhesins including the Siglec-like containing SRRPs (Takahashi et al., 2006; Pyburn et al., 2011), like GspB and Hsa (Jakubovics et al., 2005; Loimaranta et al., 2005; Takamatsu et al., 2006; Urano-Tashiro et al., 2008; Yajima et al., 2008). Hsa of *S. gordonii* DL1 and GspB of *S. gordonii* M99 bind platelet glycoprotein Ibα, MUC7 and SAG (Takahashi et al., 2002; Bensing et al., 2004; Takahashi et al., 2004; Takamatsu et al., 2006). We have identified two sialic acid binding proteins Fap1 and AsaA produced by different *S. oralis* subsp. *oralis* strains (Singh et al., 2017; Gaytán et al., 2020). Although the distribution of the genes encoding these adhesins is unknown, two of five *S. oralis* subsp. *oralis* infective isolate isolates encoded Fap1 and the remaining three AsaA.

Since *S. oralis* subsp. *oralis* strain IE12 produces sialic acid binding adhesin AsaA (Gaytán et al., 2020), the binding to SLex, demonstrated by the ability of SLex glycoconjugate to inhibit IE12 binding to mucins, was not surprising. Sialic acid is important in this interaction since we could inhibit binding to MUC7 with SLex but not Lex. We started exploring the role of *asaA* in binding to mucins using a deletion mutant; however, binding to mucins was increased with this mutant, likely due to absence of this protein increasing exposure of additional adhesins, as was previously seen for binding to fibronectin (Gaytán et al., 2021). In contrast, adherence to mucins was significantly decreased in the strain expressing AsaA lacking the non-repeat region, which contains Siglec-like domains, although some binding still remained. Although identifying the additional adhesin mechanism(s) is beyond the scope of this manuscript, the use of the *srtA* mutant provides some insights. Binding to MUC5B was virtually completely abolished by deletion of *srtA*, whereas approximately 50% of the binding remained with the *asaA* mutant, suggesting the presence of an additional Sortase A-dependent carbohydrate binding protein. Some binding to MUC7/SAG still remained in the *srtA* mutant, suggesting the presence of a Sortase A-independent mechanism of binding to this mucin. Together these data reveal that at least three mechanisms can contribute to *S. oralis* subsp. *oralis* binding to oral mucins. AsaA dependent binding is one mechanism, which appears to contribute the majority of IE12 binding to MUC7/SAG and approximately 50% of binding to MUC5B. Given the critical nature of adhesion in the oral cavity it is not surprising that these species have multiple mechanisms to mediate binding. It has previously been reported that β-1, 4-linked galactose is another receptor for *S. oralis* subsp. *oralis* on platelets (Singh et al., 2017). The fact that *S. oralis* subsp. *oralis* binding to both MUC7/SAG and MUC5B was partially inhibited by LNT, but not by LNF, and terminal galactose was present on both mucins suggests that galactose binding is a contributing factor in the interaction. However, *S. oralis* binding was strongest to MUC7/SAG despite these fractions having lower levels of galactose than MUC5B, suggesting that galactose is not the major receptor. As *S. oralis* binding mechanisms evolved for survival in the oral cavity, these data raise questions as to the role of the sialic acid binding adhesin Fap1 which was not shown to contribute to mucin binding. As the outer layer of the salivary pellicle contains MUC5B complexed with other salivary proteins (Boyd et al., 2021), it is possible that these adhesins contribute to binding to salivary components absent from the fractions examined in the current study. There have been previous reports of *S. oralis* subsp. *oralis* ATCC 10557 binding to α-amylase and unidentified low molecular weight components in saliva (Murray et al., 1992). To our knowledge, our data are the first to show that binding of *S. oralis* to salivary mucins could occur *via* Leb. The differences in both the glycoconjugates that competitively inhibit binding and the contribution of SrtA to binding suggest that different mechanisms may be involved in *S. oralis* binding to MUC5B and MUC7.

In the oral cavity, decreased salivary flow is linked to dysbiosis and increased incidence of tooth decay and oral infections (Mathews et al., 2008; Rusthen et al., 2019). It is reasonable to believe that mucin binding to oral bacteria, such as that identified in this study, decrease the amount of bacteria in the mouth since we swallow more than a liter of saliva every day. Furthermore, inflammation and infection in the mouth lead to weakened epithelial barriers, bleeding gums and bacterial access to the bloodstream. The binding of bacteria including *S. oralis* to MUC5B will result in aggregates which may also limit the ability of bacteria to enter small breaches in the barrier and limit interactions with other host molecules, similar to the decoy function mucins have been shown to have in other tissues (Linden et al., 2008a; Linden et al., 2009). Glycan epitopes that bacterial adhesins can bind to can be present on secreted mucins and on molecules attached to cell membranes of the host (Aspholm et al., 2006; Padra et al., 2018). It is thus possible that the glycan epitopes identified here also are of relevance to *S. oralis* interactions with host surfaces also in other sites of the human body, including during the development of IE.

In conclusion, *S. oralis* subsp. *oralis* bound both MUC5B and MUC7/SAG; binding to these oral mucins by IE12 is mediated by Sortase A-dependent cell wall anchored surface protein(s), including AsaA. Receptors on the mucins include Leb, LNT and SLex like glycans. These results suggest the presence of another unidentified LPXTG-containing adhesin(s) as well as a Sortase A independent binding mechanism that mediates binding of *S. oralis* subsp. *oralis* to salivary mucins. Further studies are required to elucidate the identity of these adhesins.

## Data Availability Statement

The datasets presented in this study can be found in online repositories. The raw data were uploaded on (https://glycopost.glycosmos.org/entry/GPST000163) and the annotated structures were submitted to the UniCarb-DB database (https://unicarb-dr.glycosmos.org/references/397).

## Ethics Statement

The studies involving human participants were reviewed and approved by the ethics review committee in Lund, Sweden (Dnr2021-01781). The healthy volunteers were anonymised and provided their written informed consent to participate in this study.

## Author Contributions

SL and SK designed the project. GC, SK and SL wrote the manuscript. GC, MQ-H, MG, MP and JB performed experiments. GC, MP, MQ-H and SL analyzed data. All authors contributed to the article and approved the submitted version.

## Funding

This work was supported by the Swedish research council (2019-01152), the Heart-Lung foundation (20200579), the American Heart Association (19TPA34760750), and the National Institutes of Health (R21AI149414). The funders had no role in study design, data collection and analysis, decision to publish, or preparation of the manuscript.

## Conflict of Interest

The authors declare that the research was conducted in the absence of any commercial or financial relationships that could be construed as a potential conflict of interest.

## Publisher’s Note

All claims expressed in this article are solely those of the authors and do not necessarily represent those of their affiliated organizations, or those of the publisher, the editors and the reviewers. Any product that may be evaluated in this article, or claim that may be made by its manufacturer, is not guaranteed or endorsed by the publisher.
