# Supplementary material for: Streptococcus oralis Employs Multiple Mechanisms of Salivary Mucin Binding That Differ Between Strains
Source: Front Cell Infect Microbiol. 2022 Jun 17;12:889711. doi: 10.3389/fcimb.2022.889711 (PMC9247193; doi:10.3389/fcimb.2022.889711)
Supplement: Supplementary file 1 [file Presentation_1.pdf]

Supplementary material

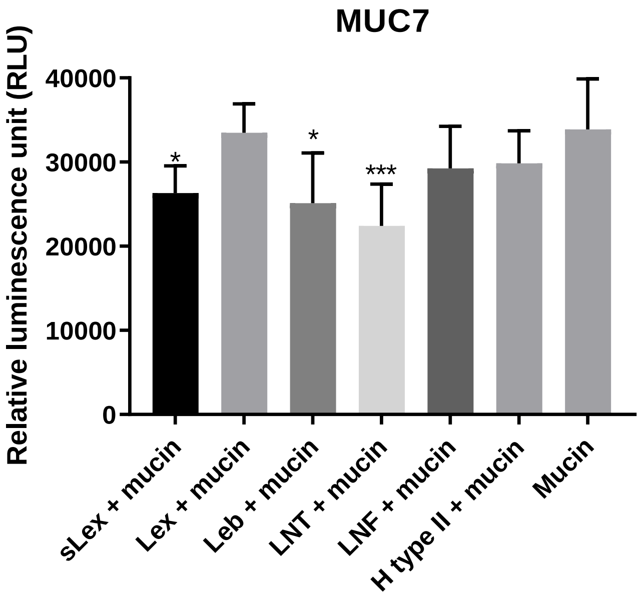

Supplementary Figure 1. Inhibition of *S. oralis* subsp. *oralis* IE12 binding to MUC7 using glycoconjugates. The MUC7/SAG rich sample from donor 2 was used in the analysis. *S. oralis* IE12 WT was pre-treated with 50 ug/mL Leb-HSA, SLex-APD-HSA, Lex-APD-HSA, LNT-APD-HSA, LNF-APD-HSA or H type 2-APD-HSA prior to use in the microtiter-based mucin binding assay. The background control was subtracted from the data shown. The bars represent mean ± SEM, ***p<0.001, *p<0.05; One-way ANOVA, Dunnett´s post hoc test.

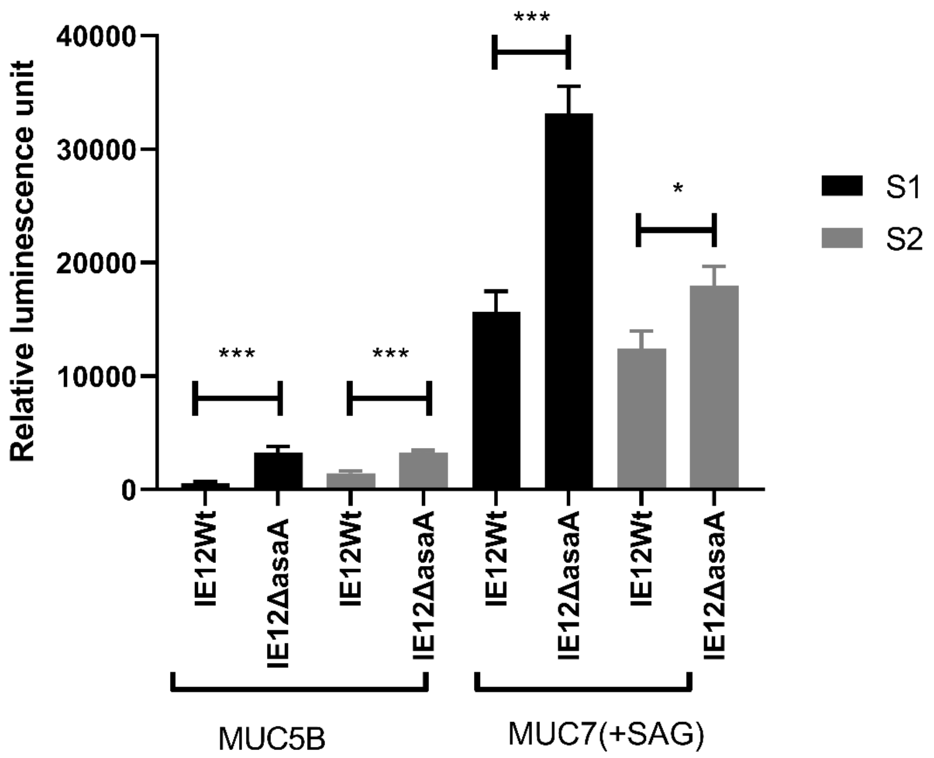

Supplementary Figure 2. Binding of *S. oralis* subsp. *oralis* IE12 WT and an isogenic *asaA* deletion mutant (IE12Δ *asaA*) to salivary mucins. IE12 WT and IE12Δ *asaA* binding to MUC5B rich and MUC7/SAG rich samples from a blood group B donor (S1) and a blood group A donor (S2) was analysed using a microtiter plate-based assay. The background control was subtracted from the data shown. The bars represent mean ± SEM, ***p<0.001, *p<0.05; Student's t-test. The results have been reproduced twice.

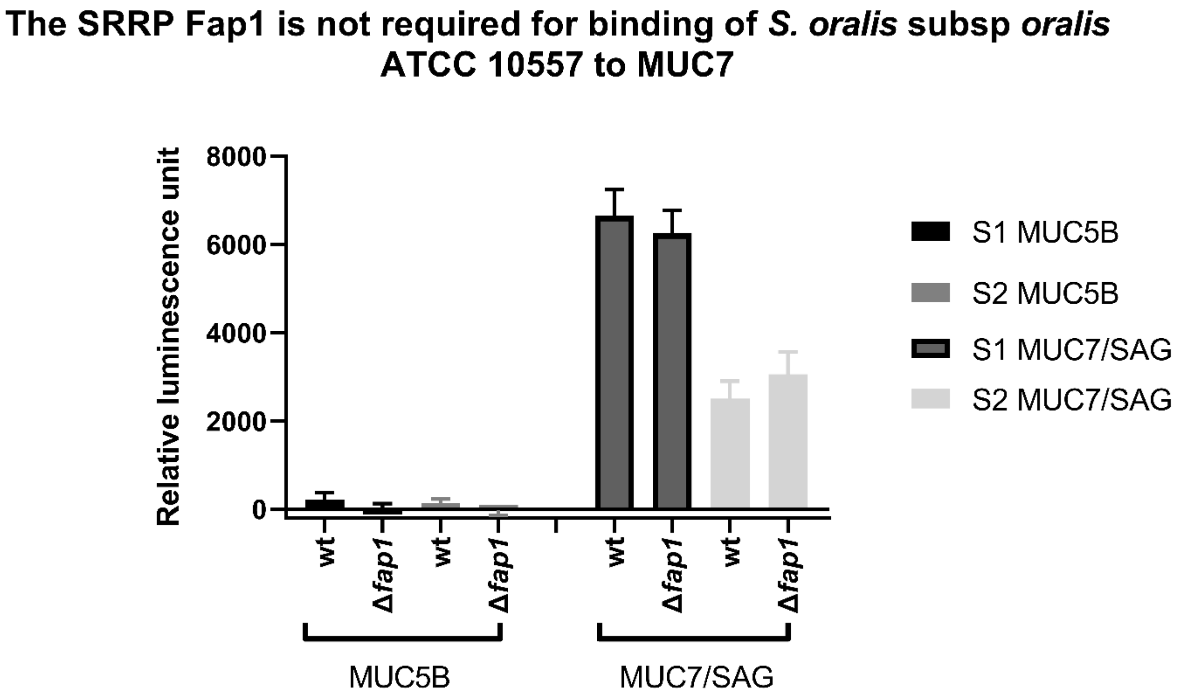

Supplementary Figure 3. Binding of *S. oralis* subsp. *oralis* ATCC 10557 WT and an isogenic *fap1* deletion mutant (ATCC 10557Δ*fap1*) to salivary mucins. ATCC 10557 WT and ATCC 10557Δ*fap1* binding to MUC5B rich and MUC7/SAG rich samples from a blood group B donor (S1) and a blood group A donor (S2) was analysed using a microtiter plate-based assay. The background control was subtracted from the data shown. The bars represent mean ± SEM (n=16). The results have been reproduced twice and data shown are the pooled results from those two experiments.
