# Supplementary material for: Streptococcus oralis Employs Multiple Mechanisms of Salivary Mucin Binding That Differ Between Strains
Source: Front Cell Infect Microbiol. 2022 Jun 17;12:889711. doi: 10.3389/fcimb.2022.889711 (PMC9247193; doi:10.3389/fcimb.2022.889711)
Supplement: Supplementary file 2 [file Table_1.docx]

|  |  | **MUC5B** | | **MUC7/SAG** | |
| --- | --- | --- | --- | --- | --- |
| **[M-H^+^]^-^** | **Supposed structure** | **Relative abundance (%)** | | **Relative abundance (%)** | |
|  |  | S1 | S2 | S1 | S2 |
| 425.3 | GlcNAcß1-3GalNAcol | 1.54 | 1.44 | 0.00 | 0.00 |
| 513.3 | NeuAcα2-6GalNAcol | 1.86 | 1.26 | 0.00 | 0.25 |
| 587.4 | Galβ1-3(GlcNAcß1-6)GalNAcol | 1.18 | 0.87 | 0.00 | 0.43 |
| 587.4 | Galβ1-4GlcNAcβ1-3GalNAcol | 2.15 | 2.50 | 1.20 | 0.44 |
| 675.4 | Galβ1-3(NeuAcα2-6)GalNAcol | 2.06 | 2.27 | 0.00 | 0.60 |
| 675.4 | NeuAcα2-3Galβ1-3GalNAcol | 1.68 | 1.78 | 27.00 | 7.21 |
| 716.4 | GlcNAcβ1-3(NeuAcα2-6)GalNAcol | 1.75 | 1.63 | 0.00 | 0.20 |
| 716.4 | GalNAcα1-3(NeuAcα2-6)GalNAcol | 0.31 | 0.61 | 0.00 | 0.16 |
| 733.4 | Fucα1-2Galβ1-3GlcNAcβ1-3GalNAcol | 1.96 | 5.32 | 0.42 | 1.23 |
| 733.4 | GalNAcα1-3(Fucα1-2)Galβ1-3GalNAcol | 0.00 | 1.68 | 0.00 | 0.62 |
| 733.4 | Fucα1-2Galβ1-4GlcNAcβ1-3GalNAcol | 0.00 | 0.99 | 0.00 | 0.29 |
| 733.4 | Fucα1-2Galβ1-3(GlcNAcβ1-6)GalNAcol | 1.87 | 1.03 | 0.29 | 0.33 |
| 749.4 | Galβ1-3(Galβ1-4GlcNAcβ1-6)GalNAcol | 2.81 | 3.37 | 1.34 | 2.98 |
| 790.5 | GlcNAcβ1-3(Galβ1-4GlcNAcβ1-6)GalNAcol | 1.93 | 1.47 | 0.00 | 0.24 |
| 813.4 | Gal-(Fuc-)(6S)GlcNAcβ1-3GalNAcol | 0.00 | 0.46 | 0.00 | 0.26 |
| 813.4 | Fucα1-2Galß1-3(6S-GlcNAcβ1-6)GalNAcol | 1.41 | 0.97 | 0.00 | 0.17 |
| 821.5 | Fucα1-2Galß1-3(NeuAcα2-6)GalNAcol | 1.75 | 1.70 | 0.40 | 0.57 |
| 829.4 | Galβ1-3(S-Galβ1-4GlcNAcβ1-6)GalNAcol | 0.38 | 1.04 | 0.00 | 0.18 |
| 829.4 | S-Gal-GlcNAcβ1-3Galβ1-3GalNAcol | 0.44 | 0.52 | 0.00 | 0.12 |
| 878.4 | GlcNAcα1-4Galβ1-3(NeuAcα2-6)GalNAcol | 0.74 | 1.00 | 0.00 | 0.31 |
| 878.4 | NeuAcα2-3Galβ1-3(GlcNAcß1-6)GalNAcol | 1.66 | 0.59 | 0.65 | 0.71 |
| 895.5 | Galβ1-3(Galβ1-4(Fucα1-3)GlcNAcβ1-6)GalNAcol | 0.00 | 0.11 | 0.70 | 0.94 |
| 895.5 | Galα1-3(Fucα1-2)Galβ1-4GlcNAcβ1-3GalNAcol | 4.08 | 0.00 | 0.77 | 0.00 |
| 895.5 | Galβ1-4(Fucα1-3)GlcNAcβ1-3Galβ1-3GalNAcol | 0.00 | 0.47 | 0.00 | 0.17 |
| 895.5 | Galα1-3(Fucα1-2)Galβ1-3(GlcNAcβ1-6)GalNAcol | 0.48 | 0.00 | 0.00 | 0.00 |
| 895.5 | Fucα1-2Galß1-4GlcNAcß1-3Galß1-3GalNAcol | 0.23 | 1.41 | 0.00 | 0.26 |
| 895.5 | Galβ1-3(Fucα1-2Galβ1-4GlcNAcβ1-6)GalNAcol | 4.65 | 5.14 | 1.20 | 1.51 |
| 936.5 | GalNAcα1-3(Fucα1-2)Galβ1-4GlcNAcβ1-3GalNAcol | 0.00 | 2.13 | 0.00 | 0.50 |
| 936.5 | GlcNAcβ1-3(Fucα1-2Galβ1-4GlcNAcβ1-6)GalNAcol | 0.00 | 0.99 | 0.00 | 0.19 |
| 952.5 | GlcNAcß1-3Galβ1-3(Galβ1-4GlcNAcβ1-6)GalNAcol | 1.41 | 1.34 | 0.18 | 0.38 |
| 966.4 | NeuAcα2-3Galβ1-3(NeuAcα2-6)GalNAcol | 0.00 | 0.17 | 3.71 | 0.90 |
| 975.4 | Fucα1-2Gal-(6S)GlcNAcβ1-3Galβ1-3GalNAcol | 0.49 | 1.00 | 0.00 | 0.13 |
| 975.4 | Fucα1-2Galß1-3(S-Gal-GlcNAcβ1-6)GalNAcol | 1.34 | 7.82 | 0.00 | 1.05 |
| 975.4 | Fucα1-2Galß1-3(Gal-(6S)GlcNAcβ1-6)GalNAcol | 1.27 | 0.00 | 0.00 | 0.00 |
| 1024.5 | Fucα1-2Galβ1-3GlcNAcβ1-3(NeuAα2-6)GalNAcol | 0.46 | 1.53 | 0.00 | 0.32 |
| 1024.5 | Fucα1-2Galβ1-4GlcNAcβ1-3(NeuAα2-6)GalNAcol | 0.21 | 1.10 | 0.00 | 0.22 |
| 1032.4 | GlcNAcα1-4Galß1-3(Gal-(6S)GlcNAcβ1-6)GalNAcol | 0.32 | 0.78 | 0.00 | 0.00 |
| 1040.5 | NeuAcα2-3Galβ1-3(Galβ1-4GlcNAcβ1-6)GalNAcol | 2.16 | 2.27 | 4.92 | 7.97 |
| 1040.5 | Galβ1-3(NeuAcα2-3Galβ1-4GlcNAcβ1-6)GalNAcol | 0.57 | 0.87 | 1.31 | 3.89 |
| 1040.5 | NeuAcα2-3Galß1-4GlcNAcß1-3Galß1-3GalNAcol | 0.33 | 0.00 | 0.00 | 0.00 |
| 1041.5 | Fucα1-2Galβ1-3(Fucα1-2Galβ1-4GlcNAcβ1-6)GalNAcol | 0.73 | 3.86 | 0.98 | 1.18 |
| 1057.5 | Galα1-3(Fucα1-2)Galβ1-4GlcNAc-Galβ1-3GalNAcol | 0.49 | 0.00 | 0.00 | 0.00 |
| 1057.5 | Galβ1-3(Galα1-3(Fucα1-2)Galβ1-4GlcNAcβ1-6)GalNAcol | 1.96 | 0.00 | 0.00 | 0.00 |
| 1057.5 | Galα1-3(Fucα1-2)Galβ1-3(Galβ1-4GlcNAcβ1-6)GalNAcol | 1.36 | 0.00 | 0.00 | 0.00 |
| 1081.5 | NeuAcα2-3Galβ1-4GlcNAcβ1-3(GlcNAcβ1-6)GalNAcol | 0.80 | 0.36 | 0.00 | 0.07 |
| 1098.5 | Galβ1-3(GalNAcα1-3(Fucα1-2)Galß1-4GlcNAcβ1-6)GalNAcol | 0.00 | 0.62 | 0.00 | 0.19 |
| 1098.5 | GlcNAcβ1-3(Galα1-3(Fucα1-2)Gal-GlcNAcβ1-6)GalNAcol | 2.10 | 0.00 | 0.00 | 0.00 |
| 1098.5 | Fucα1-2Galß1-3GlcNAcβ1-3(Galβ1-3GlcNAcβ1-6)GalNAcol | 0.69 | 1.26 | 0.00 | 0.29 |
| 1098.5 | Gal-GlcNAcβ1-3(Fucα1-2Galβ1-4GlcNAcβ1-6)GalNAcol | 0.00 | 0.62 | 0.00 | 0.15 |
| 1098.5 | Fucα1-2Galβ1-4GlcNAcβ1-3(Gal-GlcNAcβ1-6)GalNAcol | 0.00 | 0.31 | 0.00 | 0.00 |
| 1120.4 | Galß1-3(NeuAcα2-3Gal-(6S)GlcNAcβ1-6)GalNAcol | 0.34 | 0.66 | 0.00 | 0.00 |
| 1121.6 | Fucα1-2Galß1-3(Fucα1-2Gal-(6S)GlcNAcβ1-6)GalNAcol | 0.73 | 2.19 | 0.00 | 0.39 |
| 1121.6 | Fucα1-2Galß1-3(Fucα1-2Gal-(6S)GlcNAcβ1-6)GalNAcol | 0.38 | 2.56 | 0.00 | 0.51 |
| 1137.5 | Galα1-3(Fucα1-2)Galß1-3(Galß1-4(6S)GlcNAcβ1-6)GalNAcol | 0.83 | 0.00 | 0.00 | 0.00 |
| 1178.5 | (6S)GlcNAcß1-3(Galα1-3(Fucα1-2)Galß1-3GlcNAcβ1-6)GalNAcol | 0.26 | 0.80 | 0.00 | 0.24 |
| 1178.5 | Fucα1-2Galß1-4GlcNAcß1-3(Gal-(6S)GlcNAcβ1-6)GalNAcol | 0.65 | 5.40 | 0.00 | 1.04 |
| 1178.5 | Fucα1-2Galß1-4GlcNAcß1-3(Gal-(6S)GlcNAcβ1-6)GalNAcol | 0.07 | 1.10 | 0.00 | 0.18 |
| 1186.5 | Galα1-3(Fucα1-2)Galβ1-4GlcNAcβ1-3(NeuAcα2-6)GalNAcol | 0.48 | 0.00 | 0.00 | 0.00 |
| 1186.5 | Galα1-3(Fucα1-2)Galβ1-3GlcNAcβ1-3(NeuAcα2-6)GalNAcol | 1.17 | 0.00 | 0.00 | 0.00 |
| 1186.5 | Galβ1-3(NeuAcα2-3Galβ1-4(Fucα1-3)GlcNAcβ1-6)GalNAcol | 0.00 | 0.00 | 0.65 | 0.92 |
| 1186.5 | Galβ1-3(NeuAcα2-3Galβ1-3(Fucα1-4)GlcNAcβ1-6)GalNAcol | 0.00 | 0.00 | 4.14 | 4.76 |
| 1186.5 | NeuAcα2-3Galβ1-3(Fucα1-2Galβ1-4GlcNAcβ1-6)GalNAcol | 0.34 | 1.68 | 0.55 | 0.74 |
| 1186.5 | NeuAcα2-3Galβ1-3(Galβ1-4(Fucα1-3)GlcNAcβ1-6)GalNAcol | 0.33 | 0.00 | 0.00 | 0.00 |
| 1186.5 | Fucα2-3Galβ1-3(NeuAcα2-3Galβ1-4GlcNAcβ1-6)GalNAcol | 1.31 | 0.93 | 0.78 | 0.64 |
| 1187.5 | Fucα1-2Galß1-4(Fucα1-3)GlcNAcß1-3(Fucα1-2)Galß1-3GalNAcol | 0.00 | 0.30 | 0.82 | 0.74 |
| 1187.5 | Fucα1-2Galß1-3(Fucα1-2Gal-(Fuc-)GlcNAcß1-6)GalNAcol | 0.14 | 1.02 | 4.84 | 1.33 |
| 1203.5 | Galβ1-3(Galα1-3(Fucα1-2)Galβ1-4(Fucα1-3)GlcNAcβ1-6)GalNAcol | 0.23 | 0.00 | 0.00 | 0.00 |
| 1203.5 | Galα1-3(Fucα1-2)Galß1-4GlcNAcß1-3(Fucα1-2)Galß1-3GalNAcol | 0.65 | 0.00 | 0.00 | 0.00 |
| 1203.5 | Fucα1-2Galβ1-3(Galα1-3(Fucα1-2)Galβ1-4GlcNAcβ1-6)GalNAcol | 4.36 | 0.00 | 0.32 | 0.00 |
| 1227.6 | GalNAcα1-3(Fucα1-2)Galβ1-4GlcNAcβ1-3(NeuAcα2-6)GalNAcol | 0.00 | 0.39 | 0.00 | 0.07 |
| 1243.6 | NeuAcα1-3Galβ1-4GlcNAcβ1-3(Galβ1-4GlcNAcβ1-6)GalNAcol | 0.48 | 0.50 | 0.00 | 0.00 |
| 1244.6 | GlcNAcß1-3(Galα1-3(Fucα1-2)Gal-(Fuc-)GlcNAcβ1-6)GalNAcol | 0.77 | 0.00 | 0.00 | 0.00 |
| 1244.6 | Fucα1-2Galβ1-4GlcNAcß1-3(Fucα1-2Galβ1-3GlcNAcβ1-6)GalNAcol | 0.08 | 1.52 | 0.00 | 0.28 |
| 1244.6 | Fucα1-2Galβ1-3(GalNAcα1-3(Fucα1-2)Galβ1-4GlcNAcβ1-6)GalNAcol | 0.00 | 1.35 | 0.00 | 0.22 |
| 1244.6 | Fucα1-2Galβ1-4GlcNAcß1-3(Fucα1-2Galβ1-4GlcNAcβ1-6)GalNAcol | 0.00 | 0.56 | 0.00 | 0.08 |
| 1260.6 | GlcNAcα1-4Galβ1-3(Galα1-3(Fucα1-2)Galβ1-4GlcNAcβ1-6)GalNAcol | 0.52 | 0.00 | 0.00 | 0.00 |
| 1260.6 | Gal-GlcNAcβ1-3(Galα1-3(Fucα1-2)Gal-GlcNAcβ1-6)GalNAcol | 0.95 | 0.00 | 0.00 | 0.00 |
| 1260.6 | Galα1-3(Fucα1-2)Gal-GlcNAcβ1-3(Gal-GlcNAcβ1-6)GalNAcol | 0.67 | 0.00 | 0.00 | 0.00 |
| 1267.6 | Fucα1-2Galß1-3(Fucα1-2Gal-(6S)(Fuc-)GlcNAcβ1-6)GalNAcol | 0.00 | 1.10 | 0.00 | 0.00 |
| 1283.6 | Galα1-3(Fucα1-2)Galß1-3(Fucα1-2Galß1-4(6S)GlcNAcβ1-6)GalNAcol | 1.04 | 0.00 | 0.00 | 0.00 |
| 1283.6 | Fucα1-2Galß1-3(Galα1-3(Fucα1-2)Galß1-4(6S)GlcNAcβ1-6)GalNAcol | 1.05 | 0.00 | 0.00 | 0.00 |
| 1324.6 | Fucα1-2Gaβ1-4-(6S)GlcNAcβ13(Gal-(Fuc-)GlcNAcß1-6)GalNAcol | 0.25 | 1.28 | 0.00 | 0.21 |
| 1324.6 | Fucα1-2Gaβ1-4-(6S)GlcNAcβ13(Gal-(Fuc-)GlcNAcß1-6)GalNAcol | 0.04 | 0.75 | 0.00 | 0.12 |
| 1331.6 | NeuAcα2-3Galβ1-3(NeuAcα2-3Galβ1-4GlcNAcβ1-6)GalNAcol | 1.50 | 2.27 | 14.87 | 24.54 |
| 1332.6 | Galα1-3(Fucα1-2)Gal-(Fuc-)GlcNAcβ1-3(NeuAcα2-6)GalNAcol | 0.50 | 0.00 | 0.00 | 0.00 |
| 1332.6 | Fucα1-2Galβ1-3(NeuAcα2-3Gal-(Fuc-)GlcNAcβ1-6)GalNAcol | 0.43 | 0.43 | 0.63 | 0.30 |
| 1332.6 | NeuAcα2-3Galβ1-3(Fucα1-2Gal-(Fuc-)GlcNAcβ1-6)GalNAcol | 0.23 | 0.73 | 6.82 | 1.73 |
| 1332.6 | Fucα1-2Galβ1-3(NeuAcα2-3Gal-(Fuc-)GlcNAcβ1-6)GalNAcol | 0.23 | 0.26 | 1.12 | 1.09 |
| 1340.6 | Gal-(6S)GlcNAcβ1-3(Galα1-3(Fucα1-2)Gal-GlcNAcβ1-6)GalNAcol | 2.61 | 0.00 | 0.00 | 0.00 |
| 1340.6 | Galα1-3(Fucα1-2)Gal-GlcNAcβ1-3(Gal-(6S)GlcNAcβ1-6)GalNAcol | 0.60 | 0.00 | 0.00 | 0.00 |
| 1348.6 | NeuAcα2-3Galß1-3(Galα1-3(Fucα1-2)Galß1-4GlcNAcβ1-6)GalNAcol | 2.98 | 0.00 | 0.00 | 0.00 |
| 1348.6 | Galα1-3(Fucα1-2)Galß1-3(NeuAcα2-3Galß1-4GlcNAcβ1-6)GalNAcol | 0.67 | 0.00 | 0.00 | 0.00 |
| 1349.6 | Fucα1-2Galß1-3(Galα1-3(Fucα1-2)Galß1-4(Fucα1-3)GlcNAcβ1-6)GalNAcol | 3.23 | 0.00 | 0.40 | 0.00 |
| 1381.6 | S-GlcNAcα1-4Galß1-3(GalNAcα1-3(Fucα1-2)Gal-GlcNAcβ1-6)GalNAcol | 0.00 | 2.91 | 0.00 | 0.42 |
| 1389.6 | Fucα1-2Galß1-3GlcNAcβ1-3(NeuAcα2-3Galß1-4GlcNAcβ1-6)GalNAcol | 0.25 | 0.42 | 0.00 | 0.12 |
| 1389.6 | Gal-GlcNAcβ1-3(NeuAcα2-3Gal-(Fuc-)GlcNAcβ1-6)GalNAcol | 0.00 | 0.29 | 0.00 | 0.09 |
| 1389.6 | Fucα1-2Galß1-4GlcNAcβ1-3(NeuAcα2-3Galß1-4GlcNAcβ1-6)GalNAcol | 0.07 | 0.11 | 0.00 | 0.00 |
| 1406.6 | GlcNAc-Galß1-3(Galα1-3(Fucα1-2)Galß1-4(Fucα1-3)GlcNAcβ1-6)GalNAcol | 0.25 | 0.00 | 0.00 | 0.00 |
| 1406.6 | Fucα1-2Gal-GlcNAcß1-3(Galα1-3(Fucα1-2)Galß1-4GlcNAcβ1-6)GalNAcol | 1.06 | 0.00 | 0.00 | 0.00 |
| 1429.6 | Fucα1-2Galß1-3(Galα1-3(Fucα1-2)Galß1-4(6S)(Fucα1-3)GlcNAcβ1-6)GalNAcol | 0.73 | 0.00 | 0.00 | 0.00 |
| 1447.6 | Galβ1-4(Fucα1-3)GlcNAcß1-3(GalNAcα1-3(Fucα1-2)Galβ1-4GlcNAcβ1-6)GalNAcol | 0.00 | 0.82 | 0.00 | 0.00 |
| 1477.6 | NeuAcα2-3Galβ1-3(NeuAcα2-3Galβ1-4(Fucα1-3)GlcNAcβ1-6)GalNAcol | 0.19 | 0.29 | 9.16 | 9.41 |
| 1486.6 | Fucα1-2Gal-(6S)GlcNAcβ1-3(Galα1-3(Fucα1-2)Gal-GlcNAcβ1-6)GalNAcol | 1.21 | 0.00 | 0.00 | 0.00 |
| 1486.6 | Galα1-3(Fucα1-2)Gal-GlcNAcβ1-3(Fucα1-2Gal-(6S)GlcNAcβ1-6)GalNAcol | 1.06 | 0.00 | 0.00 | 0.00 |
| 1494.7 | NeuAcα2-3Galß1-3(Galα1-3(Fucα1-2)Galß1-4(Fucα1-3)GlcNAcβ1-6)GalNAcol | 2.56 | 0.00 | 0.00 | 0.00 |
| 1511.7 | Galα1-3(Fucα1-2)Galß1-3(Galα1-3(Fucα1-2)Galß1-4(Fucα1-3)GlcNAcβ1-6)GalNAcol | 1.31 | 0.00 | 0.00 | 0.00 |
| 1527.7 | S-Galβ1-4(Fucα1-3)GlcNAcß1-3(GalNAcα1-3(Fucα1-2)Galβ1-4GlcNAcβ1-6)GalNAcol | 0.00 | 0.70 | 0.00 | 0.00 |
| 1551.6 | NeuAcα2-3Gal-GlcNAcβ1-3(Galα1-3(Fucα1-2)Gal-GlcNAcβ1-6)GalNAcol | 0.81 | 0.00 | 0.00 | 0.00 |
| 1551.6 | Galα1-3(Fucα1-2)Gal-GlcNAcβ1-3(NeuAcα2-3Gal-GlcNAcβ1-6)GalNAcol | 0.24 | 0.00 | 0.00 | 0.00 |
| 1557.6 | NeuAcα2-3Galβ1-3(NeuAcα2-3Galβ1-4(Fucα1-3)(6S)GlcNAcβ1-6)GalNAcol | 0.00 | 0.00 | 0.00 | 1.29 |
| 1568.7 | Fucα1-2Gal-GlcNAcβ1-3Galß1-3(Galα1-3(Fucα1-2)Gal-GlcNAcβ1-6)GalNAcol | 1.73 | 0.00 | 0.00 | 0.00 |
| 1568.7 | Fucα1-2Gal-GlcNAcβ1-3Galß1-3(Galα1-3(Fucα1-2)Gal-GlcNAcβ1-6)GalNAcol | 0.88 | 0.00 | 0.00 | 0.00 |
| 1696.8 | NeuAcα2-3Galß1-3(NeuAcα2-3Gal-GlcNAc-Gal-GlcNAcβ1-6)GalNAcol | 0.00 | 0.00 | 0.00 | 0.41 |
| 1842.8 | NeuAcα2-3Galß1-3(NeuAcα2-3Gal-(Fuc-)GlcNAc-Gal-GlcNAcβ1-6)GalNAcol | 0.00 | 0.00 | 1.54 | 1.05 |
| 1859.8 | NeuAcα2-3Gal-GlcNAc-Galß1-3(Galα1-3(Fucα1-2)Gal-(Fuc-)GlcNAcβ1-6)GalNAcol | 0.50 | 0.00 | 0.00 | 0.00 |
| 1859.8 | NeuAcα2-3Gal-GlcNAc-Galß1-3(Galα1-3(Fucα1-2)Gal-(Fuc-)GlcNAcβ1-6)GalNAcol | 0.21 | 0.00 | 0.00 | 0.00 |
| 1876.8 | Galα1-3(Fucα1-2)Gal-(Fuc-)GlcNAcβ1-3Galß1-3(Galα1-3(Fucα1-2)Gal-GlcNAcβ1-6)GalNAcol | 1.03 | 0.00 | 0.00 | 0.00 |
| 1916.8 | NeuAcα2-3Gal-(Fuc-)GlcNAc-Gal-GlcNAc-Galß1-3(Gal-GlcNAcβ1-6)GalNAcol | 0.00 | 0.00 | 1.19 | 1.09 |
| 2022.8 | Galα1-3(Fucα1-2)Gal-(Fuc-)GlcNAcβ1-3Galß1-3(Galα1-3(Fucα1-2)Gal-(Fuc-)GlcNAcβ1-6)GalNAcol | 0.66 | 0.00 | 0.00 | 0.00 |
| 2022.8 | Galα1-3(Fucα1-2)Gal-(Fuc-)GlcNAcβ1-3Galß1-3(Galα1-3(Fucα1-2)Gal-(Fuc-)GlcNAcβ1-6)GalNAcol | 0.55 | 0.00 | 0.00 | 0.00 |
| 2061.8 | [NeuAc]2{Gal-GlcNAc-(Gal-GlcNAc-)Galß1-3(Gal-GlcNAcβ1-6)GalNAcol | 0.00 | 0.00 | 0.00 | 0.22 |
| 2061.8 | [NeuAc]2{Gal-GlcNAc-(Gal-GlcNAc-)Galß1-3(Gal-GlcNAcβ1-6)GalNAcol | 0.00 | 0.00 | 0.31 | 0.39 |
| 2061.8 | [NeuAc]2{Gal-GlcNAc-(Gal-GlcNAc-)Galß1-3(Gal-GlcNAcβ1-6)GalNAcol | 0.00 | 0.00 | 0.36 | 0.66 |
| 2207.9 | [NeuAc]2{Gal-(Fuc-)GlcNAc-(Gal-GlcNAc-)Galß1-3(Gal-GlcNAcβ1-6)GalNAcol | 0.00 | 0.00 | 0.74 | 0.68 |
| 2207.9 | [NeuAc]2{Gal-(Fuc-)GlcNAc-(Gal-GlcNAc-)Galß1-3(Gal-GlcNAcβ1-6)GalNAcol | 0.00 | 0.00 | 1.95 | 1.40 |
| 2207.9 | [NeuAc]2{Gal-(Fuc-)GlcNAc-(Gal-GlcNAc-)Galß1-3(Gal-GlcNAcβ1-6)GalNAcol | 0.00 | 0.00 | 0.45 | 0.77 |
| 2498 | NeuAcα2-3Gal-(Fuc-)GlcNAc-(NeuAcα2-3Gal-GlcNAc-)Galß1-3(NeuAcα2-3Gal-GlcNAcβ1-6)GalNAcol | 0.00 | 0.00 | 2.32 | 3.03 |
| 2498 | NeuAcα2-3Gal-(Fuc-)GlcNAc-(NeuAcα2-3Gal-GlcNAc-)Galß1-3(NeuAcα2-3Gal-GlcNAcβ1-6)GalNAcol | 0.00 | 0.00 | 0.97 | 1.22 |
